# An Unexpected Right Turn: Multipurpose A2 Catheter Causing Right Atrial Perforation

**DOI:** 10.1002/ccr3.71357

**Published:** 2025-11-02

**Authors:** Rupendra Nath Saha, Bhanu Duggal, Vijaya Kumar Varada

**Affiliations:** ^1^ AIIMS Rishikesh Rishikesh Uttarakhand India

**Keywords:** complications, mitral stenosis, PTMC, rheumatic heart disease

## Abstract

Fluoroscopic anatomy varies between patients and should be confirmed with echocardiography or angiography to avoid complications. Multipurpose A2 catheters risk cardiac perforation and must be advanced only over soft J‐tipped wires. If perforation is suspected, stabilize the catheter in place to reduce tamponade risk by wedging the site.

## Graphical abstract

Multipurpose A2 catheter induced iatrogenic right atrial perforation.

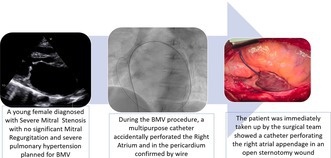

## 1 Case Image

**FIGURE 1:**
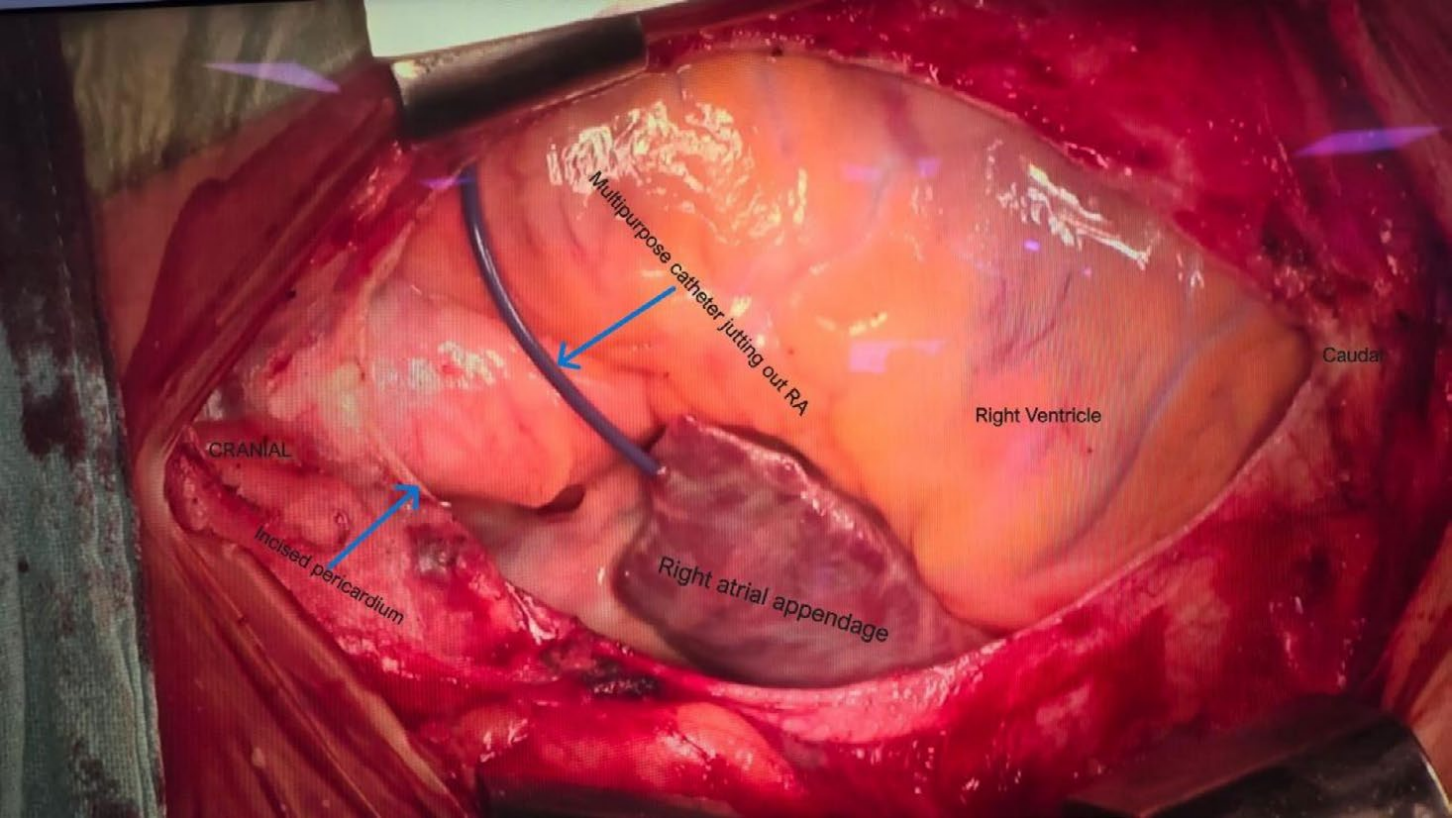
Intra‐operative open sternotomy wound showing multipurpose catheter perforating the right atrial appendage and lying in the pericardial space.

A 35‐year‐old female with no known comorbidities presented with complaints of dyspnea on exertion and intermittent palpitations for the last 6–7 months. A 2D Echo showed severe rheumatic mitral stenosis, a mitral valve area of 0.6 cm2, trace mitral regurgitation, moderate tricuspid regurgitation, and severe pulmonary hypertension (Video 1). To assess and monitor pulmonary artery systolic pressure pre‐ and post‐balloon mitral valvotomy, a multipurpose catheter is generally kept in the main pulmonary artery. The catheter was guided via the right femoral vein ➔ IVC ➔ RA ➔ RV ➔ pulmonary artery (Video 2). However, since the pulmonary artery pressure tracings could not be retrieved in the hemodynamic monitor, and on aspiration, straw‐colored pericardial fluid was aspirated, immediately a Terumo wire was inserted, and it was found to be coiled in the pericardial space (Video 3). The aspiration from the catheter also revealed pericardial fluid. A 2D echocardiography confirmed no tamponade. The patient was immediately shifted to emergency cardiothoracic vascular OT, and open sternotomy revealed a catheter tip entering the right atrial free wall (Figure 1). The catheter was removed, and the right atrial free wall rent and the mitral valve were repaired. The patient was successfully discharged on medications with stable vitals. The patient was symptomatically better till follow‐up after three months. Echocardiography review showed PASP of 27 mmHg.

**VIDEO 1:** TTE PLAX view showing rheumatic mitral stenosis with enlarged left atrium and enlarged right ventricle. Video content can be viewed at https://onlinelibrary.wiley.com/doi/10.1002/ccr3.71357.

**VIDEO 2:** Fluoroscopy view showing multipurpose catheter from the right atrium advanced in the direction of the pulmonary artery, white arrow shows the ideal path it should have followed. Video content can be viewed at https://onlinelibrary.wiley.com/doi/10.1002/ccr3.71357.

**VIDEO 3:** Fluoroscopy view showing wire from the multipurpose catheter in the pericardial space. Video content can be viewed at https://onlinelibrary.wiley.com/doi/10.1002/ccr3.71357.

## 2 Discussion

BMV is the first line for the management of severe rheumatic mitral stenosis. In this procedure, under light sedation, right heart catheterization is performed for studying pre‐ and post‐BMV. Most of the complications reported with the procedure are cardiac tamponades due to inadvertent punctures of the left atrium/pericardium with the Brokenborough needle or significant mitral regurgitation [1]. The right atrium is rarely involved.

In our case, fluoroscopy showed the multipurpose catheter was in the apparent position of the pulmonary artery; such an anomalous position was thought later to be due to an enlarged or a malpositioned right atrial appendage or a misdirected diagnostic catheter in retrospect. There are several reasons for improper pressure tracing, such as improper tubing, lack of zeroing, improper residual contrast in the measurement system, kinking of catheters, and clotting in catheters. If one encounters such improper tracings, first, the catheter should be aspirated with a syringe to see the contents or aspirate any clot. If after multiple aspirations, there is no proper aspirate, ideally, the wire should not be passed as it increases the risk of clot embolization, especially in left heart catheterization. In our cases, as straw‐colored fluid had already been aspirated, a soft hydrophilic Terumo wire was passed to confirm the diagnosis. In case of any doubt, a right atrial angiography would delineate the right atrial appendage and reduce complications [2]. Lastly, multipurpose A2 catheters are commonly used for right and left heart catheterization and generally considered safe for manipulations. These diagnostic catheters are rarely reported to cause cardiac perforations, as in a case where the Tiger catheter was responsible for a left ventricular perforation [3]. Thus, all diagnostic catheters should always be advanced with the help of a hydrophilic, J‐tipped wire for all catheterization procedures.

## Author Contributions

**Rupendra Nath Saha:** conceptualization. **Bhanu Duggal:** resources. **Vijaya Kumar Varada:** methodology.

## Disclosure

The authors have nothing to report.

## Ethics Statement

The Cardiology Departmental Ethics Committee of AIIMS Rishikesh approved the project. In addition, we confirm that all applicable rules and guidelines are executed in all processes. Informed consent was obtained from all subjects. All data were collected following sound clinical practice principles and the Declaration of Helsinki.

## Consent

Written informed consent was obtained from the patient to publish this report in accordance with the journal's patient consent policy. We confirm that the manuscript has been read and approved by all named authors and that no other persons have satisfied the criteria for authorship but are not listed. We further confirm that all have approved the order of authors noted in the manuscript. We confirm that we have given that there are no objections to publication, including the timing of publication, concerning intellectual property.

## Data Availability

The data supporting this study's findings are not openly available due to reasons of sensitivity and are available from the corresponding author upon reasonable request. Data are located in controlled access data storage at the Cardiology Department of AIIMS Rishikesh.
